# Cross-platform dataset of multiplex fluorescent cellular object image annotations

**DOI:** 10.1038/s41597-023-02108-z

**Published:** 2023-04-07

**Authors:** Nathaniel Aleynick, Yanyun Li, Yubin Xie, Mianlei Zhang, Andrew Posner, Lev Roshal, Dana Pe’er, Rami S. Vanguri, Travis J. Hollmann

**Affiliations:** 1 Department of Pathology and Laboratory Medicine, Memorial Sloan Kettering Cancer Center, New York, NY USA; 2 Program for Computational and Systems Biology, Sloan Kettering Institute, Memorial Sloan Kettering Cancer Center, New York, NY USA; 3 Howard Hughes Medical Institute, New York, NY USA; 4 Present Address: Department of Pathology and Laboratory Medicine, Children’s Hospital of Philadelphia, Philadelphia, PA USA; 5 Present Address: Bristol Meyers Squibb, Princeton, NJ USA

**Keywords:** Image processing, Fluorescence imaging

## Abstract

Defining cellular and subcellular structures in images, referred to as cell segmentation, is an outstanding obstacle to scalable single-cell analysis of multiplex imaging data. While advances in machine learning-based segmentation have led to potentially robust solutions, such algorithms typically rely on large amounts of example annotations, known as training data. Datasets consisting of annotations which are thoroughly assessed for quality are rarely released to the public. As a result, there is a lack of widely available, annotated data suitable for benchmarking and algorithm development. To address this unmet need, we release 105,774 primarily oncological cellular annotations concentrating on tumor and immune cells using over 40 antibody markers spanning three fluorescent imaging platforms, over a dozen tissue types and across various cellular morphologies. We use readily available annotation techniques to provide a modifiable community data set with the goal of advancing cellular segmentation for the greater imaging community.

## Background & Summary

Understanding tissue cellular architecture is essential for the characterization and prognostication of disease. Immunohistochemistry (IHC), often used in clinical diagnostic and research workflows, is a technique which allows for the spatially resolved evaluation of, most commonly, a single biomarker. Multiplexed cellular imaging methods similarly allow spatial evaluation of, most commonly, protein expression by single cells in tissue samples^1–3^ with higher marker content and phenotypic determinations. The accurate assessment of cell-based biomarker profiles relies on delineating cellular boundaries, known as cell segmentation, such that the biomarker detection can be attributed to the correct cell. Furthermore, delineating protein expression within cellular compartments can be biologically important (i.e. translocation from cytoplasm to nucleus). While machine learning-based algorithms have shown some success for automating cellular segmentation^4^, vast amounts of accurate cell annotations are required to train these algorithms, which can be tedious and costly to obtain. While there are publicly available annotated nuclei within hematoxylin and eosin slides^5^, analogous multiplex fluorescent imaging datasets are rare.

**Fig. 1:**
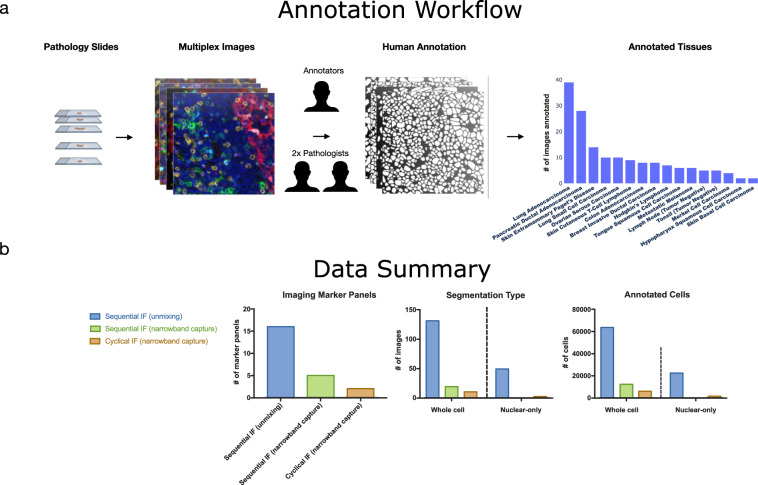
(**A**) Cellular annotation is performed starting from pathology slides which have been stained for a variety of markers with multiplexed imaging platforms. The images are then annotated and reviewed by 2 pathologists to create consensus annotations. (**B**) Data summary stratified by multiplexed imaging platform.

We fill this gap by providing manual cell annotations, which are modifiable, across many cancer types spanning solid tumor and hematolymphoid as well as normal lymph node and tonsil tissue (Fig. 1). We provide annotations spanning 3 imaging platforms and over 40 antibody markers to assist in building and benchmarking segmentation algorithms robust against multiplex staining and detection methods (Table 1). Both whole cell and nuclear annotations are provided to allow for more granular segmentation of cellular compartments. To account for variation in imaging platforms, we provide annotations on tissue acquired via sequential immunofluorescence (IF) with unmixing (via Akoya Vectra 3.0), sequential IF with narrowband capture (via Ultivue InSituPlex with Zeiss Axioscan image capture) and cyclical IF with narrowband capture (via Akoya CODEX). While advantages and disadvantages of these platforms are described elsewhere^6^, differences in staining and scanning technologies can introduce small morphological differences in digitized images which segmentation algorithms need to be robust against.

**Table 1:** Markers and annotated tissue types, stratified by detection method.

Detection (Commercial Platform)	Markers	Tissue types
Sequential IF unmixing (Akoya Vectra 3.0)	ARG-1, CD11b, CD138, CD163, CD20, CD3, CD30, CD4, CD40, CD40L, CD66b, CD68, CD8, CK7, CTLA4, Dapi, FoxP3, GITR, GZMB, ICOS, IDO, Ki67, LAG3, MHC-I, MHC- II, MUM1, P63, PanCK, PAX8, PD-1, PD-L1, PD-L2, TCF1, TOX, Vista	Lung adenocarcinoma, extramammary Paget disease, pancreatic ductal adenocarcinoma, lung small cell carcinoma, colon adenocarcinoma, Hodgkin lymphoma, breast ductal carcinoma, serous ovarian carcinoma, squamous cell carcinoma, Merkel cell carcinoma, squamous mucosa
Sequential IF narrowband capture (Ultivue InSituPlex + Zeiss Axioscan)	CD3, CD4, CD68, CD8, Dapi, FoxP3, PanCK, PD-L1, Sox10	Cutaneous T-cell lymphoma, pancreatic adenocarcinoma, basal cell carcinoma, melanoma
Cyclical IF narrowband capture (Akoya CODEX)	CD20, CD21, CD31, CD3, CD4, CD45RO, CD8, Dapi, HLA-DR, PanCK	Lymph node, tonsil

**Fig. 2:**
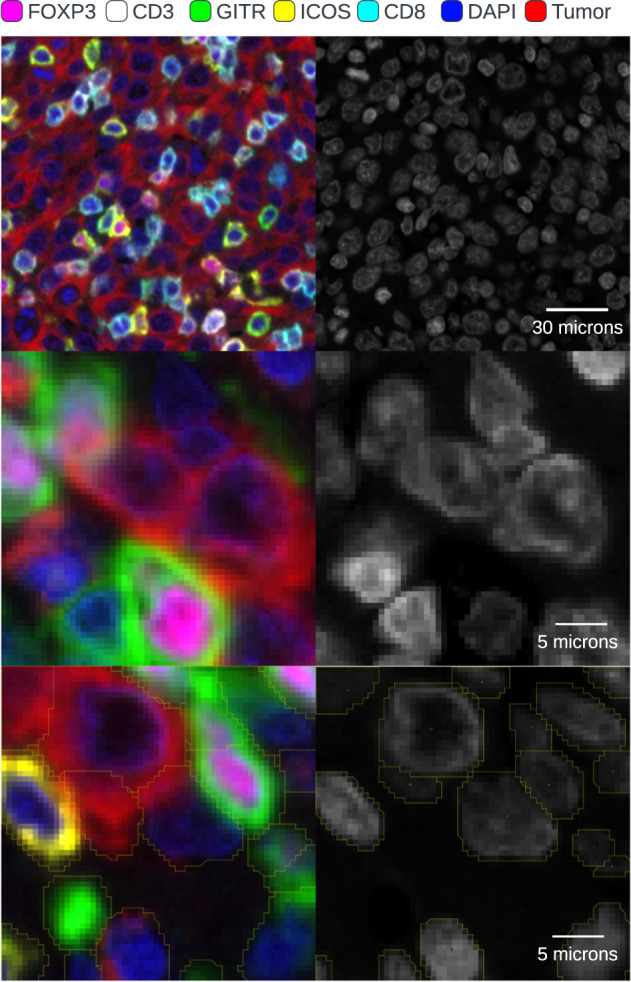
Example image area from lung adenocarcinoma tissue including: multichannel image and corresponding nuclear channel (top), example of overlapping cells with corresponding nuclear channel (middle) and example of overlapping nuclei with corresponding annotated nuclear channel (bottom).

In creating a cellular annotation dataset, consistency must be maintained. A typical cross section of tissue is the extraction of a 2-dimensional tissue section at a particular thickness from a 3-dimensional tissue volume. As a result, cells are not consistently sectioned in a particular orientation or plane. Additionally, a section could potentially encompass two cells which are overlapping or fragments of cells or cell processes thereby leading to partial cell representations and overlapping cells (Fig. 2).

We present a set of adaptable manual annotations which have been individually assessed for quality and accuracy by several experts. We consistently handle overlapping cells by ensuring that annotations do not have boundaries which overlap. Cell objects consist of instances of whole cell annotations (82,058) or nuclei-only annotations (23,716). A subset of the whole cell annotations contain separate matching nuclear annotations performed on the same image crop. We provide manual and adaptable whole cell and nuclear object annotations across platforms and markers using publicly available annotation software as a resource which can be used to further develop, refine and evaluate cellular segmentation algorithms (Table 2). We also provide corresponding parent images from which the annotated crops are derived.

**Table 2:** Marker panels, number of annotated images and objects stratified by detection method.

Detection (Commercial Platform)	Micron/Pixel Size (magnification)	Number of Panels	Number of Images Annotated	Total Number of Objects in Multichannel Images	Total Number of Objects in Nuclear-only Images
Sequential IF unmixing (Akoya Vectra 3.0)	0.5 (20X)	16	131 Multichannel 49 Nuclear-only	63,714	22,358
Sequential IF narrowband capture (Ultivue InSituPlex + Zeiss Axioscan)	0.325 (20X)	5	19 Multichannel	12,425	0
Cyclical IF narrowband capture (Akoya CODEX)	0.3774 (20X)	2	10 Multichannel 2 Nuclear-only	5,919	1,358

## Methods

All tissue samples were derived from a combination of published^7–9^ and unpublished studies performed at Memorial Sloan Kettering Cancer Center which were approved by the institutional review board (16-1683, 15-021, 22-274). While lung adenocarcinoma and pancreatic ductal carcinoma were prioritized tissue types as part of the Human Tumor Atlas Network initiative, we included a diversity of organ systems to maximize generalizability and usability. All tissue samples were formalin fixed and paraffin embedded by conventional surgical pathology guidelines at MSKCC. Annotations were performed in imaging areas where cells could be clearly delineated with minimal obfuscations and where all markers were represented with low background. In cancer specimens, cells were annotated within representative tumor and stromal areas.

We annotate across three multiplex imaging platforms: Sequential IF with unmixing, Sequential IF with narrowband capture and Cyclical IF with narrowband capture.

1. **Sequential Multiplex IF unmixing (Akoya Vectra 3.0)**
  The Vectra 3.0 tissue staining protocol is described in previous work^7,10^. Markers for each of the 16 distinct panels are specified in the metadata within the data repository.
2. **Sequential Multiplex IF narrowband capture (Ultivue InSituPlex with Zeiss Axioscan image capture)**
  We utilized the Ultivue InSituPlex technology to perform specimen staining followed by scanning with a Zeiss Axioscan. Reagent assay and slide preparation was performed according to the description of Ultivue ready to use protocol. The 4 µm FFPE tissue sections were baked for 1 hour at 62 degrees Celsius in vertical slide orientation with subsequent prestaining with deparaffinization and 20 minutes of antigen retrieval with Leica Bond ER2 using the Leica Bond RX automated research stainer. Slides were then incubated with four DNA barcode- conjugated primary antibodies (Table 3) using 20 minutes incubation. Once the slide was incubated with the primary antibody, the DNA barcodes of each target were elongated. Fluorescent probes that are complementary to the elongated barcodes were then added to the sample to bind and label each round of targets. Each target within a single imaging round is labeled with a spectrally distinct fluorophore to enable multiplexed whole slide imaging without the need for spectral unmixing. Nuclear counterstain was performed with the reagent inside the kit. The slides were mounted with ProLong Gold antifade reagent mounting medium (Invitrogen P36930). The full slide imaging was acquired with a Zeiss Axioscan at 20X (200X final magnification) which was analyzed and annotated using ImageJ.

**Table 3:** Markers included in Ultivue ready to use protocol.

DNA Barcode Conjugated Antibody	Clone ID	Detection Channel	Part number
CD8	C8/144B	FITC	ULT00139
CD68	KP-1	Cy3	ULT00137
PD-L1	73-10	Cy5	ULT00142
panCK	AE1/1E3	Cy7	ULT00159
3. **Cyclical Multiplex IF narrowband capture (Akoya CODEX)**

This technique uses oligo conjugated primary antibodies with cyclical detection by fluorescent-dye labeled complementary strands. The 7 µm thickness FFPE tissue samples were mounted to the polylysine (Sigma, P8920-500ML) pre-treated coverslip (EMS, 72204-01) and air dried for 24 to 48 hours. The tissue coverslip was baked at 56 °C for 20 minutes followed by the hydration process and antigen retrieval with DAKO Target Retrieval Solution, pH9 (S2367) with a pressure cooker for 20 min. The oligonucleotides barcode pre-conjugated antibody cocktails assay was applied on the tissue coverslip and incubated for 3 hours at room temperature. The stained tissue coverslip and the 96 well plate with fluorescent signals detectable complementary strands to the parent oligonucleotide were loaded to the imaging stage. The cyclical imaging was automatically acquired with an inverted fluorescent microscope (Keyence, BZ-810) at 20x with each cycle detecting 3 primary barcoded antibodies at a time in DAPI, Cy3, Cy5 and Cy7 channel and then with stripping of the complementary strand using Dimethyl sulfoxide (DSMO, Sigma, 472301-4 L) before applying the next cycle of complementary detection strands. Once all cycles are complete, the individual scans were stacked together into a single multiplex QP tiff image which was imported into ImageJ for analysis and annotation.

### Annotation

**Fig. 3:**
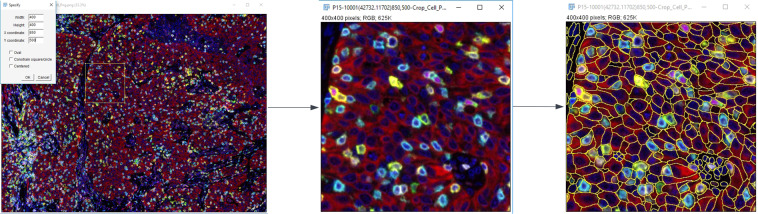
ImageJ workflow for cropping and annotation. In this example (left), starting from a parent image (lung adenocarcinoma shown), an area of representative tumor with sufficient markers being expressed is chosen (middle), and individual whole cells are annotated (right).

Cellular annotations were manually created using ImageJ (Fig. 3). Each image is a 400 × 400 or 800 × 800 pixel crop from a larger multiplex image. Annotations in each image were made with the freehand tool and added to the ROI manager. Each annotation is a cellular object representing either: (1) a nucleus and its associated cytoplasmic or membranous staining, (2) a nucleus with no cytoplasmic or membranous staining or (3) cytoplasmic or membranous staining with no visible nuclei. Each cell was annotated such that no two annotations have overlapping boundaries (Fig. 2). Each image was annotated by at least 2 and up to 3 annotators. The annotations were reviewed by a pathologist and consensus annotations were finalized.

## Data Records

All data records, including images and a spreadsheet containing metadata which maps the tissue type and markers, are contained in synapse^11^ (10.7303/SYN27624812). Annotations correspond to:

16 separate panels acquired using Sequential IF with unmixing (Akoya Vectra 3.0)

5 separate panels acquired using Sequential IF with narrowband capture (Ultivue InSituPlex with Zeiss Axioscan image capture)

2 separate panels using 13-color Cyclical IF narrowband capture (Akoya CODEX)

A total of 131 full cell annotation sets and 49 nuclear annotation sets from Sequential IF with unmixing, 10 full cell annotation sets and 2 nuclear annotation sets from Cyclical IF and 19 full cell annotation sets from Sequential IF with narrowband capture are provided. The accompanying metadata describes the color assignments to the markers within their respective panels. Nuclear/whole cell masks, corresponding image crops and parent images from which the crops were extracted are provided.

The directory structure follows, where “coordinates of crop” indicates the top left corner of the parent image that the patch is taken from. In the case of the Sequential Multiplex IF Imaging with narrowband capture, the size of the images necessitated 2 crops which is indicated by the 2 sets of coordinates.

**Sequential Multiplex IF Staining with unmixing (Akoya Vectra 3.0):**

Vectra/Panel Number/panel number – Sample ID[Coordinates of crop]

**Sequential Multiplex IF Imaging with narrowband capture (Ultivue InSituPlex with Zeiss Axioscan):**

Zeiss/Sample ID[Coordinates of initial crop][Coordinates of second crop]

**Cyclical Multiplex IF Imaging Acquisition with narrowband capture (Akoya CODEX):**

Codex/Sample ID [Coordinates of crop]

## Technical Validation

Imaging was performed on specimens where correlative monoplex DAB staining was performed and only images with adequate sensitivity and specificity were annotated. Individual annotations were validated via consensus amongst 2–3 annotators and pathologist approval.

## Data Availability

Annotations were manually performed using ImageJ, there is no accompanying code as the software is publicly available (ImageJ 1.52p).
